# Green Tea Polyphenol (-)-Epicatechin Pretreatment Mitigates Hepatic Steatosis in an In Vitro MASLD Model

**DOI:** 10.3390/cimb46080531

**Published:** 2024-08-16

**Authors:** Marija Hefer, Ana Petrovic, Lucija Kuna Roguljic, Tea Omanovic Kolaric, Tomislav Kizivat, Catherine H. Wu, Ashraf A. Tabll, Robert Smolic, Aleksandar Vcev, Martina Smolic

**Affiliations:** 1 Department of Translational Medicine, Faculty of Dental Medicine and Health Osijek, J. J. Strossmayer University of Osijek, 31000 Osijek, Croatia; mhefer@fdmz.hr (M.H.); anapetrovic@fdmz.hr (A.P.); lkuna@fdmz.hr (L.K.R.); cwu@uchc.edu (C.H.W.); aa.tabll@nrc.sci.eg (A.A.T.); 2 Department of Pharmacology and Biochemistry, Faculty of Dental Medicine and Health Osijek, J. J. Strossmayer University of Osijek, 31000 Osijek, Croatia; tomanovic@fdmz.hr (T.O.K.); rsmolic@fdmz.hr (R.S.); 3 Department of Nuclear Medicine and Oncology, Faculty of Medicine Osijek, J. J. Strossmayer University of Osijek, 31000 Osijek, Croatia; tkizivat@mefos.hr; 4 Department of Integrative Medicine, Faculty of Dental Medicine and Health Osijek, J. J. Strossmayer University of Osijek, 31000 Osijek, Croatia; avcev@fdmz.hr

**Keywords:** MASLD, NAFLD, pretreatment, polyphenols, (-)-epicatechin

## Abstract

**Abstract:** Metabolic dysfunction-associated steatotic liver disease (MASLD), previously known as non-alcoholic fatty liver disease (NAFLD), is becoming more prominent globally due to an increase in the prevalence of obesity, dyslipidemia, and type 2 diabetes. A great deal of studies have proposed potential treatments for MASLD, with few of them demonstrating promising results. The aim of this study was to investigate the potential effects of (-)-epicatechin (EPI) on the development of MASLD in an in vitro model using the HepG2 cell line by determining the metabolic viability of the cells and the levels of PPARα, PPARγ, and GSH. HepG2 cells were pretreated with 10, 30, 50, and 100 μM EPI for 4 h to assess the potential effects of EPI on lipid metabolism. A MASLD cell culture model was established using HepG2 hepatocytes which were exposed to 1.5 mM oleic acid (OA) for 24 h. Moreover, colorimetric MTS assay was used in order to determine the metabolic viability of the cells, PPARα and PPARγ protein levels were determined using enzyme-linked immunosorbent assay (ELISA), and lipid accumulation was visualized using the Oil Red O Staining method. Also, the levels of intracellular glutathione (GSH) were measured to determine the level of oxidative stress. EPI was shown to increase the metabolic viability of the cells treated with OA. The metabolic viability of HepG2 cells, after 24 h incubation with OA, was significantly decreased, with a metabolic viability of 71%, compared to the cells pretreated with EPI, where the metabolic viability was 74–86% with respect to the concentration of EPI used in the experiment. Furthermore, the levels of PPARα, PPARγ, and GSH exhibited a decrease in response to increasing EPI concentrations. Pretreatment with EPI has demonstrated a great effect on the levels of PPARα, PPARγ, and GSH in vitro. Therefore, considering that EPI mediates lipid metabolism in MASLD, it should be considered a promising hepatoprotective agent in future research.

## 1. Introduction

Metabolic dysfunction-associated steatotic liver disease (MASLD) is a condition marked by hepatic steatosis with potential progression to cirrhosis and liver cancer. MASLD manifests as a multisystem disease, impacting organs and regulatory pathways, thereby elevating risks of type 2 diabetes, cardiovascular diseases, and even chronic kidney disease. Linked closely with metabolic disorders such as dyslipidemia, hypertension, and hyperglycemia, MASLD presents as a growing public health challenge [1,2,3,4,5,6].

The onset of MASLD is closely linked to the accumulation of lipids, lipotoxicity, and the consequent oxidative stress [7,8,9]. Oxidative stress has a central role in both the onset and progression of MASLD where the disturbance in lipid metabolism is one of the main causes for the accumulation of fat in hepatocytes and the generation of reactive oxygen species (ROS) [10,11,12,13].

Fatty changes in MASLD arise from various genetic factors and de novo lipogenesis, which involves the down-regulation and up-regulation of a wide range of lipogenic transcription factors such as peroxisome proliferator-activated receptors: PPARα and PPARγ [14,15,16,17]. PPARs are known to regulate adipogenesis and lipid storage in both physiological and pathological states: PPARα mostly regulates fatty acid transport and beta-oxidation, whereas PPARγ predominantly regulates fat storage [18,19,20,21].

Moreover, when considering the ROS-generating processes in MASLD, glutathione (GSH), along with superoxide dismutase (SOD), is considered to be one the most important factors in ROS scavenging. A few pathological states in the liver have been shown to include GSH as one of the key antioxidants that controls inflammation, apoptosis, and disease progression. Both GSH and SOD are known to be increased in MASLD, which indicates that the levels of GSH and SOD can be used to predict the course of the disease and its progression [8,22,23,24].

To this date, there are several proposed treatments for fatty liver disease which mainly include diet regulation, controlling the intake of fats and sugars, both hyperglycemia and hyperlipidemia, and increasing physical activity. However, none of these have shown significant results when it comes to the clinical outcome and prognosis of the disease. Antioxidant compounds represent a promising therapeutic approach when considering hepatic steatosis in general, as they are known to exert various beneficial effects, including their ability to modulate both lipogenesis and lipid catabolism [25,26,27,28,29,30].

In recent years, polyphenol-rich foods emerged as a potential therapeutic option for the inhibition of tumor proliferation, regulation of enzymes involved in redox reactions, and various metabolic conditions, including metabolic syndrome, atherosclerosis and MASLD. Polyphenols are reported to have hepatoprotective, antioxidant, and anti-inflammatory properties, thereby exhibiting a high potential in ameliorating the effects of MASLD in vitro, in vivo, and in some clinical studies, as well. The evolving understanding of polyphenols highlights their potential role beyond ROS scavenging, emphasizing their broad antioxidant properties and their impact on various biological pathways [31,32,33,34,35,36].

Polyphenols have been reported to influence de novo lipogenesis and fatty acid beta-oxidation by affecting the activity of lipogenic enzymes and enhancing the activity of some lipolytic proteins [33,37,38]. Due to their diverse biological effects and antioxidant properties, we examined one of the green tea polyphenols (-)-epicatechin (EPI) and its effect on MASLD.

Green tea contains biologically active compounds and is well known for its anti-inflammatory and anticancer properties, with catechins serving as its primary antioxidant agents. Experimental evidence suggests that the catechins found in green tea play a vital role in MASLD development and progression by reducing the concentration of ROS, modulating energy homeostasis and inflammatory responses. Along with epigallocatechin, epicatechin gallate, and epigallocatechin gallate (EGCG), EPI is also one of the catechins found in green tea [39,40,41,42,43,44].

EPI demonstrates relatively high bioavailability, with 95% absorption indicated by urinary excretion and circulation through the body as various phase II metabolites with the half-life of EPI being up to 4 h. EPI was found to alleviate CCl_4_-induced hepatotoxicity and oxidative stress in vivo, highlighting its potential for safer and more effective liver protection compared to other green tea catechins [45,46,47]. Additionally, EPI has been proven effective in attenuating cardiometabolic risk factors such as dyslipidemia and obesity [48].

Considering its beneficial effects in cardiometabolic disorders, as well as studies reporting its hepatotoxicity-reducing properties in vivo, the aim of this study was to investigate EPI’s hepatotoxicity and lipid accumulation ameliorating effects in MASLD using an in vitro model. Green tea catechin doses typically range from 25 to 750 mg per serving, with daily intakes between 25 mg and 1500 mg, provided mostly in capsules and green tea extracts [49]. Concentrations of 10 and 30 µM of EPI, corresponding to doses up to 1500 mg from tea extracts or capsules, were used to test EPI’s efficacy [50]. To test potential hepatotoxicity, concentrations of 50 and 100 µM EPI were used.

## 2. Materials and Methods

### 2.1. HepG2 Cell Culturing

The HepG2 cells (ATCC, Manassas, VA, USA) were grown in Dulbecco’s Modified Eagle’s Medium (DMEM, low glucose) supplemented with 1% penicillin/streptomycin solution (PS) and 10% Fetal Bovine Serum (FBS). Prior to the experiments, HepG2 cells were incubated in multiwell plates at 37 °C (5% CO_2_) for 24 h in DMEM (low glucose) supplemented with 1% PS without FBS.

### 2.2. Pretreatment with (-)-Epicatechin and Treatment with OA

Cells were pretreated with 10, 30, 50, and 100 µM solutions of (-)-epicatechin (EPI/Sigma-Aldrich, St. Louis, MO, USA) for different time periods (most of the conducted experiments include a 4 h-pretreatment, while 2-, 8-, and 24 h-pretreatments were used initially). After the pretreatment, EPI solutions were completely removed from the cells and the cells were treated with oleic acid (OA) for 24 h to induce steatosis. Untreated cells (UT) group contains medium with 0.6% FBS (Fetal Bovine Serum) and 0.9% ethanol which was used to dissolve the OA. EPI used in experiments was dissolved in a medium containing 0.07% ethanol. The EPI control group (EPIc) contains cells pretreated with 100 µM EPI and is used to determine the effect of EPI alone on HepG2 cells. After the pretreatment, in the EPIc group, the medium containing 0.07% ethanol and EPI was replaced by solvent used in the UT group. UT was used as a negative control, while OA was used as a positive control.

### 2.3. MTS Assay

MTS assay is a colorimetric method used for determining the cell metabolic viability. The experiment was performed in a 96-well plate to determine the proper concentration of OA and the duration of pretreatment. Following the experiments, 20 µL of MTS reagent (Promega, Madison, WI, USA) was added and the cells were incubated for 2 h. The measurement of absorbance was performed at a wavelength of 490 nm using a Microplate Reader.

### 2.4. ELISA

Elisa Kits (FineTest^®^, Wuhan, China) were used for the determination of PPARα and PPARγ—both experiments were conducted according to the manufacturer’s instructions using 6-well plates for the incubation and (pre)treatment of the cells.

### 2.5. Oil Red O Staining

The experiment was conducted in a 24-well plate for the visualization of lipid accumulation. The cells were washed with Phosphate Buffered Saline (PBS) and incubated in 10% formalin solution for 1 h at 4 °C. Fixed cells were then washed with 60% isopropyl alcohol. Oil Red O solution (Sigma-Aldrich, St. Louis, MO, USA) was added to each well and the cells were incubated for 30 min. The cells were once again washed with 60% isopropyl alcohol and the fatty changes were visualized using a microscope. Microscopic observation was conducted at 40× magnification and the pictures were acquired using the Motic Images Plus 3.0 software.

#### Oil Red O Quantification

To quantify Oil Red O, the cells were incubated in 250 µL of 99% isopropyl alcohol for 5 min on a tilting board. The resulting solutions containing the extracted Oil Red O were transferred to a 96-well plate, and the absorbance was measured at 518 nm, as described [51].

### 2.6. Measuring Intracellular GSH Levels

A Glutathione Assay kit (Sigma-Aldrich, St. Louis, MO, USA) was used for the experiment; the cells were washed with PBS and suspended at a cell density of 10^6^ cells/mL. PBS was removed and 5% 5-Sulfosalicylic Acid (SSA) was added to a cell suspension. The cell suspension was frozen and thawed several times, and the resulting supernatant was used to determine the concentration of GSH in each of the given samples according to the manufacturer’s instructions.

### 2.7. Statistical Analysis

One-way and Two-way ANOVA were used to determine the statistical significance. The results were expressed using the mean values for each group, where we compared the mean value of OA alone with the mean values of cells pretreated with EPI and the untreated cells (UT).

## 3. Results

### 3.1. Establishment of an In Vitro Model of MASLD

**Figure 1:**
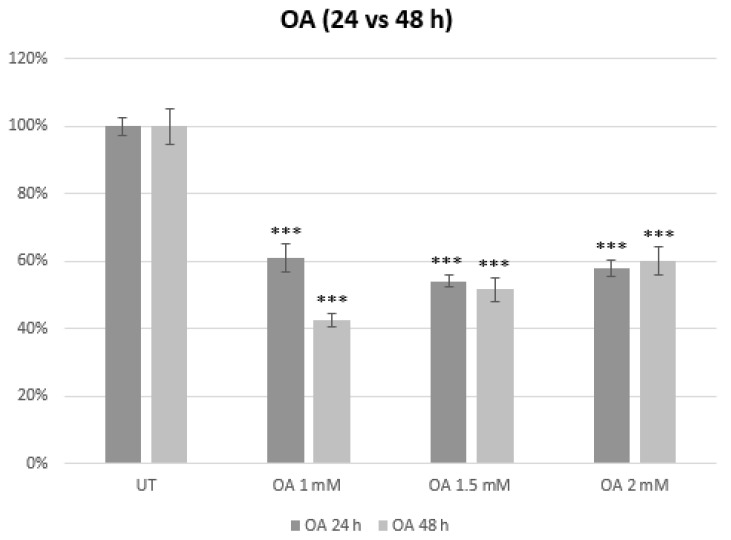
Determination of the metabolic viability by MTS assay after exposure to varying concentrations of OA and different time periods in the HepG2 cells. The measurement of absorbance was performed at a wavelength of 490 nm. UT was used as the negative control and the results were shown as a percentage of metabolic viability relative to UT (100%). At least six replicates were used and the bars assigned with the following asterisks were shown to be statistically significantly different from UT (*** *p* < 0.001). UT: Untreated cells, OA: Oleic Acid.

Three different concentrations of OA were used (1, 1.5, and 2 mM) to determine the difference in the toxicity of OA over 24 and 48 h of treatment (Figure 1). The metabolic viability of the cells was found to decrease up to 54.1% in cells treated with 1.5 mM OA after 24 h of treatment, similar to the metabolic viability after a 48 h treatment with 1.5 mM OA where the metabolic viability decreased up to 51.6% compared to the UT group (*** *p* < 0.001). All of the concentrations showed a significant impact on the metabolic viability and, from the given MTS results, 1.5 mM OA (24 h) was selected for an in vitro model of MASLD in HepG2 cells.

Based on the MTS assay results, the 1.5 mM OA treatment for 24 h was chosen as the optimal condition for developing an in vitro model of MASLD in HepG2 cells. This concentration provided a substantial and consistent reduction in cell viability during 24 and 48 h treatments, thereby making it suitable for studying the effects of OA-induced toxicity in this model.

### 3.2. EPI Pretreatment and Subsequent Oleic Acid Exposure in HepG2 Cells

**Figure 2:**
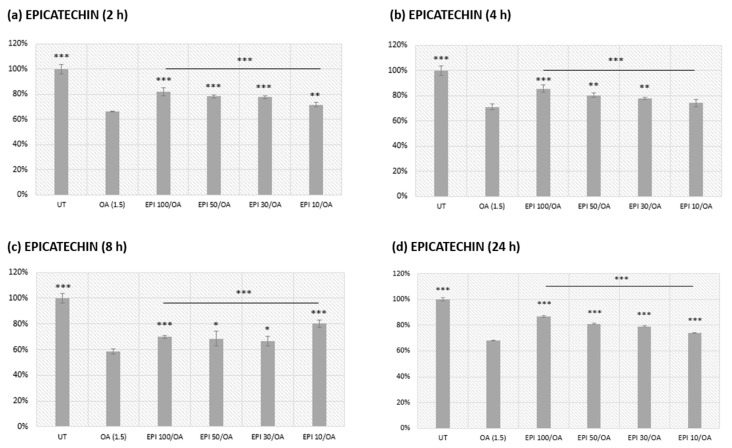
Determination of the metabolic viability by MTS assay after pretreatment with varying concentrations of EPI and treatment with 1.5 mM OA in the HepG2 cells. The measurement of absorbance was performed at a wavelength of 490 nm. OA was used as the positive control and the results were shown as a percentage of metabolic viability relative to UT (100%). UT: untreated cells, EPI 100, 50, 30, and 10/OA correspond to the groups pretreated with the given concentrations of EPI + 24 h treatment with OA. At least five replicates were used and the bars assigned with the following asterisks were shown to be statistically significantly different from OA alone (*** *p* < 0.001, ** *p* < 0.01, and * *p* < 0.05).

Cells were pretreated with 10, 30, 50, and 100 µM solutions of EPI for different durations (2, 4, 8, and 24 hpretreatments) and the cells were incubated in 1.5 mM OA solution for 24 h (Figure 2). 4 and 24 h pretreatments with EPI showed similar improvements in metabolic viability of the cells treated with OA compared to the OA alone (*** *p* < 0.001).

The cell metabolic viability was 82.5, 77.4, 75.1, and 71.5% for EPI 100, 50, 30, and 10/OA, respectively, in a 4 h pretreatment. Similarly, the cell metabolic viability was 86.8, 81.1, 79.1, and 74.1% for EPI 100, 50, 30, and 10/OA, respectively, in a 24 h pretreatment. Considering that the half-life of EPI is up to 4 h, this could be a potential indicator of why 4 h and 24 h pretreatments had similar effects on the cell metabolic viability. A 2 h pretreatment also had similar effects, but it was not utilized in the experiments succeeding the MTS assay because the half-life of EPI is reached upon 4 h of treatment. Therefore, we selected a 4 h pretreatment with EPI, based on its half-life, to determine whether a short pretreatment could hinder the progression of MASLD in HepG2 cells.

#### Assessment of a 4 h EPI Pretreatment Alone on HepG2 Cell Metabolic Viability

**Figure 3:**
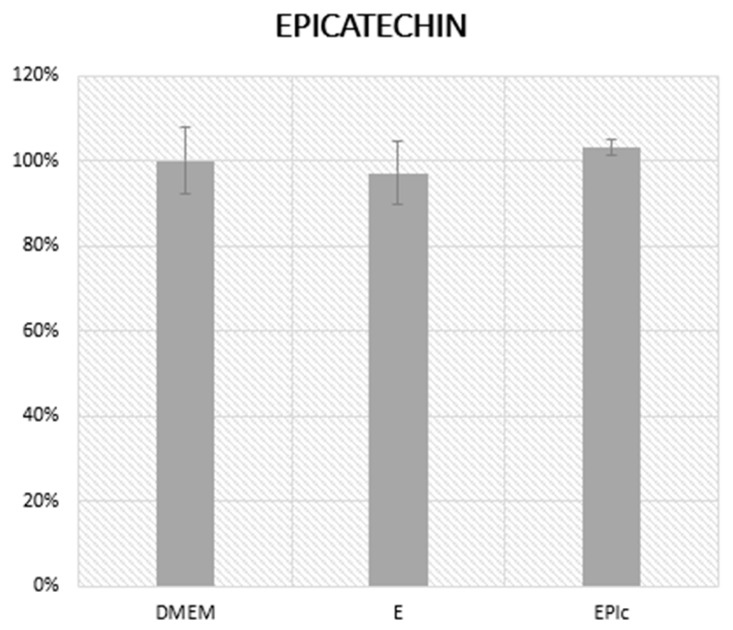
Determination of the metabolic viability by MTS assay after pretreatment with 100 µM EPI (EPIc). The measurement of absorbance was performed at a wavelength of 490 nm. DMEM was used as the negative control and the results were shown as a percentage of metabolic viability relative to DMEM (100%). DMEM: Dulbecco’s Modified Eagle’s Medium, E: medium with 0.07% ethanol, EPIc: 100 µM EPI. At least four replicates were used and no results were shown to be statistically significantly different from DMEM alone.

Cells were pretreated with the highest EPI concentration used in experiments, 100 µM solution of EPI, for 4 h to examine the effect of EPI on the metabolic viability of HepG2 cells (Figure 3). An additional group containing medium with 0.07% ethanol (E) used to dissolve EPI was utilized in this experiment, along with a DMEM-only group which was used as a negative control.

The results suggest that neither the ethanol control (E) nor the EPI-treated group (EPIc) showed statistically significant differences from the DMEM-only group. This confirms that the low concentration of ethanol (0.07%) used to dissolve EPI does not affect the metabolic viability of HepG2 cells. Moreover, high concentrations of EPI (100 µM) do not adversely affect the metabolic viability of HepG2 cells when compared to the untreated control (DMEM). This indicates that EPI, at the concentration used, is not cytotoxic to HepG2 cells. Therefore, EPI can be considered suitable for use in experiments following MTS, without concerns about compromising HepG2 metabolic viability.

### 3.3. Peroxisome Proliferator-Activated Receptors: PPARα and PPARγ

**Figure 4:**
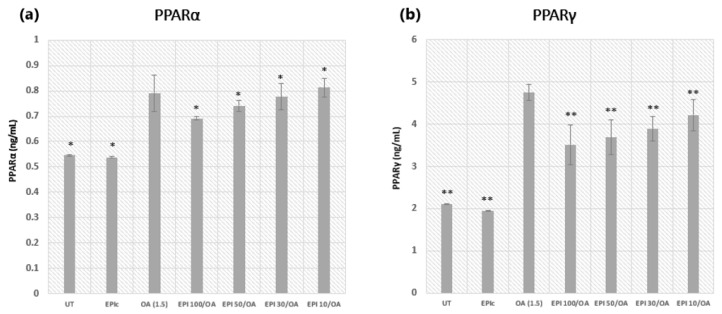
Determination of the PPARα and PPARγ levels in HepG2 cells after pretreatment with varying concentrations of EPI and treatment with 1.5 mM OA. Subfigure (**a**) shows the levels of PPARα, while subfigure (**b**) shows the levels of PPARγ. The measurement of absorbance was performed at a wavelength of 450 nm. OA was used as the negative control and the results were represented as a concentration of both PPARs in ng/mL. At least three replicates were used in two separate experiments and the bars assigned with the following asterisks were shown to be statistically significantly different from OA alone: * *p* < 0.05 for the PPARα and ** *p* < 0.01 for PPARγ groups.

Cells were pretreated with 10, 30, 50, and 100 µM solutions of EPI for 4 h and the cells were incubated in 1.5 mM OA solution for 24 h to determine the concentration of PPARα and PPARγ in HepG2 cells. OA alone caused a significant increase in the levels of PPARα and PPARγ compared to the UT group, which proves that OA induces a change in the lipid metabolism of OA-treated HepG2 cells. Both experiments showed a decrease in the levels of PPARs with an increasing concentration of EPI used as a pretreatment, as shown in Figure 4 (* *p* < 0.05, ** *p* < 0.01). However, the lowest concentration (EPI 10) proved to be ineffective (Figure 4a) as it exhibited a higher level of PPARα than the OA group, while there was a significant decrease in the levels of PPARγ (Figure 4b). According to the results, the highest concentration (EPI 100) proved to be the most effective in the prevention of OA-induced increase in the protein levels of both PPARs.

EPI pretreatment resulted in a dose-dependent decrease in the levels of PPARα and PPARγ. The lowest concentration of EPI (10 µM) was ineffective in reducing PPARα levels, exhibiting even higher levels than the OA group. However, it significantly decreased PPARγ levels. The highest concentration of EPI (100 µM) was the most effective, significantly reducing the OA-induced increase in the protein levels of both PPARα and PPARγ.

Therefore, pretreatment with EPI, particularly at the highest concentration (100 µM), could effectively counteract the OA-induced elevation of PPARα and PPARγ levels in HepG2 cells. This suggests that EPI can modulate lipid metabolism alterations induced by OA, with potential implications for therapeutic strategies targeting MASLD. The results also indicate that EPI alone group (EPIc) did not exhibit significant changes in PPARα and PPARγ levels in HepG2 cells when compared to UT.

### 3.4. Visualization of Fatty Changes and ORO Quantification in HepG2 Cells

**Figure 5:**
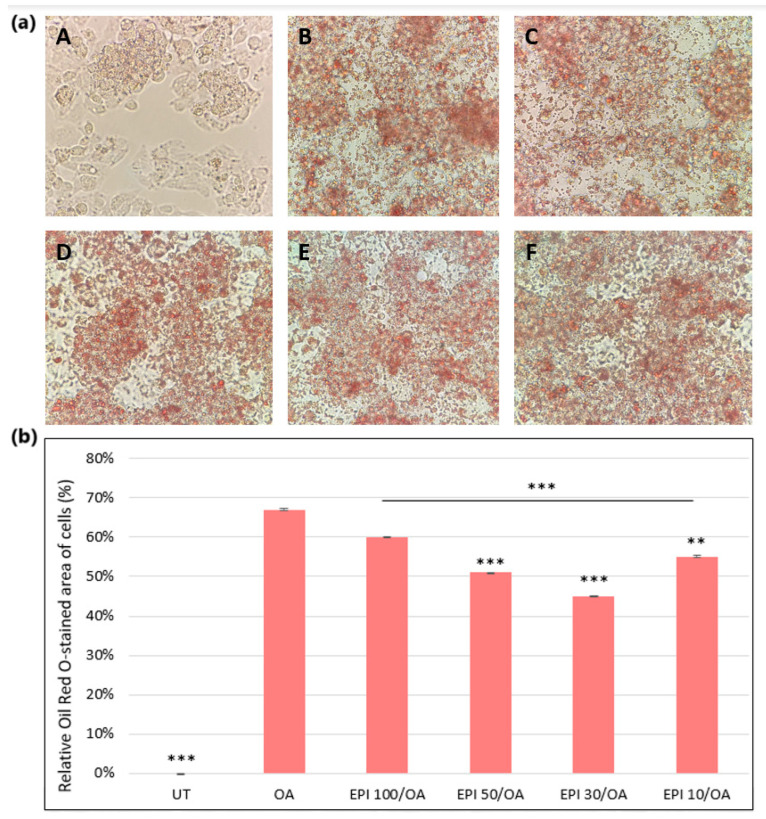
(**a**) The effect of varying concentrations of EPI on the development of OA-induced steatotic changes. A—UT, B—OA, C—EPI100/OA, D—EPI50/OA, E—EPI30/OA, F—EPI10/OA. At least four replicates were used from two separate experiments. The images were acquired with a 40× objective lens using Motic Images Plus software. (**b**) Oil Red O quantification. The results were shown as a relative Oil Red O-stained area of the cells, where the UT group was set at 0%. At least four replicates were used and the bars assigned with the following asterisks were shown to be statistically significantly different from OA alone (*** *p* < 0.001, ** *p* < 0.01).

The UT group showed a healthy epithelial-like morphology of HepG2 cells, while the cells treated with OA alone showed a distortion in the morphology with a high number of lipid droplets (Figure 5a). The cells pretreated with EPI demonstrated a decrease in the number of lipid droplets with a mild improvement in cell morphology. However, the morphology of the pretreated cells was still significantly distorted in the EPI100/OA group, with a slight improvement in the morphology seen in groups EPI50/OA, EPI30/OA, and EPI10/OA. These images might suggest that a dose of 100 µM EPI may not be as efficient as lower concentrations of EPI used in the experiment.

The experiment showed that EPI has a concentration-dependent effect on mitigating OA-induced steatotic changes in HepG2 cells. While the highest concentration of EPI (100 µM) resulted in some reduction of lipid droplets, it did not significantly improve cell morphology and lipid accumulation, compared to the lower concentrations. The lower concentrations of EPI (50 µM, 30 µM, and 10 µM) were more effective, with EPI30/OA showing the best results in terms of reducing lipid accumulation and improving cell morphology (Figure 5a,b).

### 3.5. Intracellular GSH Concentration

**Figure 6:**
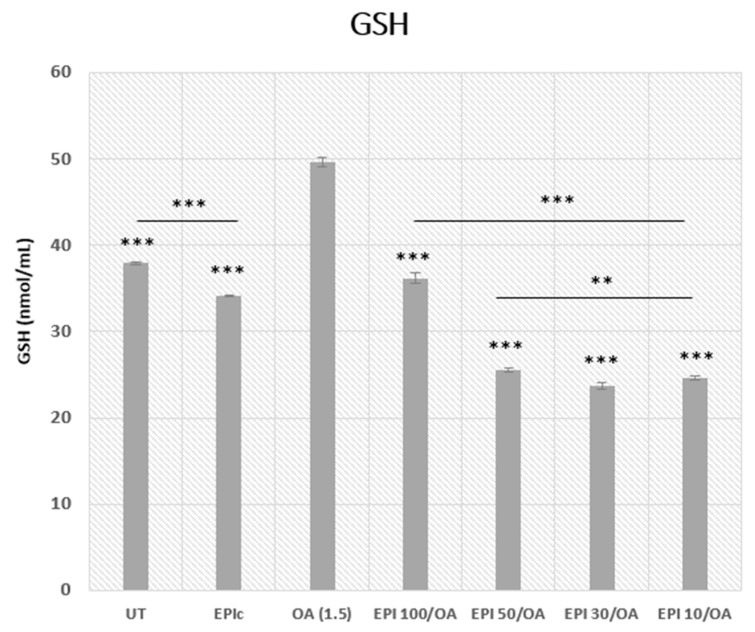
Determination of intracellular GSH levels in HepG2 cells after pretreatment with varying concentrations of EPI and treatment with 1.5 mM OA. The measurement of absorbance was performed at a wavelength of 415 nm. OA was used as the negative control and the results were represented as a concentration of GSH in nmol/mL. At least three replicates were used in two separate experiments and the bars assigned with the following asterisks were shown to be statistically significantly different from OA alone (*** *p* < 0.001 and ** *p* < 0.01).

Intracellular GSH levels were measured in UT, EPIc, and OA, as well as the cells pretreated with 4 h EPI + 24 h OA. The results showed an increase in the level of GSH in cells treated with OA only compared to the UT group, which is indicative of OA-induced oxidative stress. Introduction of EPI as a pretreatment inhibited the OA-induced generation of GSH in a concentration-dependent manner, where the lowest concentrations were proved to have the greatest impact on the intracellular GSH levels as shown in Figure 6 (*** *p* < 0.001, ** *p* < 0.01).

EPI pretreatment before OA exposure reduces the OA-induced GSH increase in a concentration-dependent manner. Lower concentrations of EPI are more effective, which could suggest that EPI helps mitigate oxidative stress, thereby decreasing the GSH upregulation caused by OA exposure. The significant decrease in GSH levels in the EPI-only group (EPIc) compared to the UT and OA groups shows that EPI might influence GSH metabolism by downregulating the synthesis of GSH or enhancing the utilization of GSH in antioxidant defense mechanisms, thereby reducing its intracellular levels.

## 4. Discussion

Our study revealed a concentration-dependent decrease in metabolic viability, with 1.5 mM OA exhibiting the most pronounced effect after both 24 and 48 h. This concentration was chosen for our experiments as it significantly impacted the metabolic viability of HepG2 cells with minimal difference in the metabolic viability between 24 and 48 h of treatment, thereby providing a foundation for an in vitro model of MASLD. Here we also referred to similar works that were done on Huh7 cells using 0.5 and 1 mM OA to induce Non-Alcoholic Steatosis (NAS) and on HepG2 cells by using 1.5 mM OA to induce MASLD. The results obtained while using an MTS assay on Huh7 cells and 1 mM concentration of OA were similar to our results [52,53].

EPI pretreatment demonstrated a concentration-dependent improvement in metabolic viability of the cells, with a significant improvement observed at 4 and 24 h pretreatments. The 2 and 8 h pretreatments showed slight variations, where the 2 h pretreatment showed significantly different results from the results obtained by the 4 and 24 h pretreatments. On the other hand, 8 h pretreatment did not show a concentration-dependent increase in the metabolic viability of the cells, so it was excluded as a possible option for the pretreatment of HepG2 cells. 

The 4 h pretreatment was selected for our experiments in order to evaluate the effectiveness of a shorter pretreatment period. Considering that most of the research done was conducted using 10 µM solutions of polyphenols and a 24 h cotreatment, we chose different concentrations and a preatreatment to determine whether EPI could prevent the progression of MASLD in a dose-dependent manner [54,55,56]. Also, the pretreatment was chosen to eliminate the possibility of interaction between the polyphenol we used and the OA. Here, the results of an MTS assay suggested a potential protective effect of EPI against OA-induced cytotoxicity in a dose-dependent manner.

Furthermore, examination of PPARα and PPARγ levels following EPI pretreatment revealed a decrease in the levels of both PPARs with increasing EPI concentrations. This is probably due to the ability of polyphenols to activate 5′-adenosine monophosphate activated protein kinase (AMPK) which in turn inhibits PPARα and PPARγ [57,58,59,60,61,62].

However, other studies that used polyphenols such as EGCG and resveratrol for the treatment of MASLD showed a decrease in the levels of PPARγ in treated groups, while there was an increase in the levels of PPARα when a lower concentration of polyphenols was used in vitro (10 µM). Unexpectedly, the lowest EPI concentration (EPI 10) we used exhibited a higher level of PPARα than the OA group, while a significant decrease in PPARγ levels was noted using the same concentration. This result would be consistent with the results obtained in other studies using the same concentration of polyphenols [53,63,64].

This difference between our results and the results of other studies, when determining whether polyphenols cause a decrease or an increase in the levels of PPARα, requires further exploration to elucidate the interactions between EPI and PPARs in the context of MASLD when using higher concentrations of polyphenols with respect to the type of treatment used. 

Morphological alterations in HepG2 cells treated with OA were evident, including a high number of lipid droplets. EPI pretreatment demonstrated a reduction in lipid droplets, indicating a potential ameliorative effect on the lipid accumulation associated with MASLD. This was reported by other studies, as well [65,66,67]. However, the observed improvement remained mild, suggesting the need for additional strategies to fully restore cell morphology. In addition to this, the potential ineffectiveness of 100 µM EPI in reducing lipid accumulation should also be evaluated.

OA induced steatosis in HepG2 cells and caused an increase in the intracellular GSH levels, which is indicative of an increase in the concentration of ROS. As was previously demonstrated by other studies, elevated GSH levels induced by OA suggest an increase in the levels of oxidative stress [68,69].

EPI pretreatment effectively inhibited OA-induced GSH generation in a dose-dependent manner, with lower EPI concentrations demonstrating the most significant impact. This implies a potential antioxidative property of EPI, highlighting its role in mitigating oxidative stress associated with MASLD. Other studies that included the effect of polyphenols on GSH levels showed different results, where an increase in the GSH production caused by polyphenols is proposed to be a result of the nuclear factor erythroid 2-related factor 2 (NRF2) pathway activation. NRF2 is involved in the regulation of genes involved in the regulation of cellular oxidation and signaling [22,70,71,72,73].

Polyphenols such as EGCG, resveratrol, catechin, and quercetin are reported to increase GSH levels, while chyrisin and apigenin showed a decrease in the levels of GSH in cells other than HepG2 and only hydroxychalcones were reported to cause GSH depletion in HepG2 cells [68,74,75,76,77,78,79].

Considering that there is limited work done on HepG2 cells regarding the effect of polyphenols on intracellular GSH levels, further research is required to elucidate the role of polyphenols in the GSH activation pathway.

Collectively, our results demonstrate a significant impact of EPI on the OA-induced steatotic changes in vitro, and key molecular pathways implicated in MASLD progression. The concentration-dependent responses should be taken into account when considering the (pre)treatment options for MASLD. Moreover, the unexpected PPARα response requires further exploration regarding EPI’s influence on specific aspects of lipid metabolism when considering concentrations higher than 10 µM or even consider using some other polyphenols such as EGCG.

Further research is required when taking into account the limited number of studies conducted both in vitro and in vivo when considering the effect of polyphenols on the progression of MASLD. The unexpected PPARα response could be additionally confirmed using other biomarkers in alternative cell lines, or conducting in vivo studies. Furthermore, determining the effects of EPI in a pretreatment of MASLD when considering the activation of AMPK and/or NRF2 is crucial for the elucidation of the potential role of EPI in the PPARα and GSH signaling pathways. Metabolic diseases, especially those modulated by PPARs, are known to exhibit significant sex-based differences in their response to polyphenols [80]. Therefore, future research should also include the investigation of the potential sex-dependent effects of epicatechin and other green tea polyphenols.

## 5. Conclusions

In conclusion, our study contributes valuable insights into the effect of EPI on the progression of MASLD in an in vitro model using HepG2 cells, demonstrating the potential of EPI in mitigating OA-induced cytotoxicity and oxidative stress. Therefore, further investigations involving pretreatment with other polyphenols are required to clarify the specific molecular mechanisms underlying the observed concentration-dependent responses, thereby providing a solid foundation for future therapeutic strategies against MASLD.

## Data Availability

The data presented in this study are available on request from the corresponding authors.
